# Attributes of atherosclerotic plaques in carotid artery disease: a Doppler ultrasound assessment

**DOI:** 10.1590/1677-5449.202401862

**Published:** 2025-10-27

**Authors:** Dyana Carolina Teixeira Trevisan, Carla Aparecida Faccio Bosnardo

**Affiliations:** 1 São Leopoldo Mandic de Araras – SLMA, Araras, SP, Brasil.

**Keywords:** Doppler ultrasonography, carotid artery diseases, atherosclerotic plaque

## Abstract

Atherosclerosis is a chronic inflammation of the artery wall caused by injury to the vascular endothelium and influenced by many different risk factors. Carotid stenosis is a crucial marker of cardiovascular risk that is especially relevant among men over the age of 65. Treatment and diagnostic methods for carotid disease are the subject of debate. This integrative review analyses research on Doppler ultrasonography assessment of atherosclerotic plaques. Searches of the PubMed and SciELO databases identified 69 articles, 16 of which provided details of this method of analysis. Doppler ultrasound has become the technique most often used because it is non-invasive and provides detailed information on plaque morphology and blood flow. Doppler ultrasonography enables comprehensive evaluation of plaques, identifying their morphological characteristics and estimating the risk of complications. It is concluded that Doppler ultrasonography is essential for assessment of carotid disease, enabling a noninvasive and detailed assessment and contributing to early identification of high-risk patients and improving clinical results.

## INTRODUCTION

Atherosclerosis is a condition characterized by chronic and reparative inflammation of the artery wall in response to injury to the vascular endothelium and is influenced by multiple risk factors, such as genetics, age, sex, hyperlipidemia, arterial hypertension, and smoking.^1^ Under normal conditions, the arterial endothelium plays a significant role in protection of blood vessels. However, injuries can occur in response to traumatic conditions, provoking a pathological process that results in accumulation of lipids within a fibrous capsule adhering to the artery wall, giving rise to an atherosclerotic plaque. As the disease progresses, the plaque may undergo a process of calcification.^1,2^

These atherosclerotic plaques may be at risk of rupture, with a likelihood of formation of thrombi, which in turn can reach and occlude other blood vessels, potentially leading to complications such as stroke if the carotid arteries are involved. Depending on the site at which the thrombus becomes lodged, an ischemic stroke may result, particularly if the thrombus has originated in unstable plaques in the internal carotid, which feeds the brain directly.^2,3^

Carotid stenosis is an important marker of cardiovascular risk, with variable prevalence in the population, contributing significantly to cases of cerebral ischemia. The condition tends to affect men more than women, especially men aged over 65 years.^2-5^

Treatment of carotid atherosclerotic disease is the subject of continuous discussion because of its considerable clinical importance and there are constant theoretical and technological developments. In addition to treatment options, there is also debate about diagnostic methods and their influence on medical decision making. In carotid stenosis cases, computed tomography angiography and Doppler ultrasound are common options used to evaluate plaques.^4^

While it does provide detailed information, computed tomography angiography is an invasive procedure that is expensive and requires use of iodinated contrast, which is contraindicated for some patients. In contrast, Doppler ultrasound is noninvasive, accessible, and safe, yielding important information on anatomy and blood flow, without exposure to ionizing radiation or contrast, and is suitable for expectant mothers and allergic patients, for example.^6^

Considering the above, this article presents an integrative review conducted with the objective of highlighting the advantages of using Doppler ultrasound to assess atherosclerotic plaques. The primary objective is to present supporting evidence to contribute to analysis and classification of the features of atherosclerotic plaques identified using Doppler ultrasonography, compiling data on how this approach can be effective for clinical decision-making and for defining therapeutic management.

## METHODS

An integrative literature review was conducted focusing on characterization of atherosclerotic plaques in carotid disease using Doppler ultrasonographic assessment. The study sought to answer the following research question: “Which characteristics of atherosclerotic plaques are detected by arterial Doppler ultrasonography in patients with carotid disease and how do these characteristics influence the risk of rupture, ischemia, and occurrence of stroke?”

The PICO strategy (patient/intervention/control/outcome) was used to resolve the question. Searches were run on PubMed, Cochrane Library, Biblioteca Virtual em Saúde (BVS) and SciELO databases using Medical Subject Headings (MeSH)/Health Science Descriptors (DeCs). Search terms were combined using the Boolean operators AND and OR to maximize sensitivity, as follows: (Echocardiography, Doppler OR Doppler Ultrasonography) AND (Carotid Artery Diseases OR Arterial Diseases OR Carotid Artery Atherosclerosis) AND (Atheroma OR Atherosclerotic Plaque) AND (Diagnostic Evaluation OR Diagnostic Imaging OR Ultrasonographic Evaluation).

The search was also run in Portuguese on the SciELO database using the following strategy: ((ultrassonografia Doppler OR duplex scan) AND (carótidas OR carótida OR doenças arteriais OR aterosclerose das carótidas)) AND ((plaque de ateroma OR plaque aterosclerótica) AND (avaliação diagnóstica OR diagnóstico por imagem OR avaliação ultrassonográfica)).

The review included work published from 2018 to 2024 in Portuguese or English that dealt specifically with vascular Doppler ultrasonography of the carotids and its associations with atherosclerosis. Incomplete articles, theses, dissertations, and studies that were not directly related to the research question were excluded.

A standardized table was constructed to organize the data, containing the following information: authors, article title, year of publication, type of study, objectives, analytical parameters, and main findings, in addition to an analysis of evidence levels using the Oxford system. Studies were organized in the table in the order in which they are cited in the discussion in the present article. A critical analysis was conducted of the selected articles, based on reading them in minute detail, supporting discussion of their findings and answering the study’s research question.

Two reviewers selected studies independently by reading titles and abstracts. Selected articles were then read in full and discussed. In cases of differences of opinion, a third reviewer was consulted to take the final decision.

Research Ethics Committee approval was waived for this project because it is exclusively based on data publicly available on databases and involves no direct human participants.

All of the data were organized as shown in Table 1, which lists all of the clinical studies selected and used to compile this integrative literature review and demonstrate the importance of studying atherosclerotic plaques with arterial Doppler ultrasonography to prevent ischemic events such as stroke.

**Table 1:** Summary of selected clinical studies and reviews used to compile the present integrative review of the literature, supporting the importance of studying atherosclerotic plaques using arterial Doppler ultrasonography to prevent ischemic events such as stroke.

**Title**	**Author and year of publication**	**Objectives and analytic parameters**	**Results**	**Oxford evidence level**
Prediction of Stroke Risk by Detection of Hemorrhage in Carotid Plaques: Meta-Analysis of Individual Patient Data	Schindler et al.,^7^ 2020	Cohort study with 560 patients evaluating the risk of stroke in carotid stenosis cases, considering presence of intraplaque hemorrhage on magnetic resonance.	Intraplaque hemorrhage, which is more common in symptomatic carotid stenosis, increases the risk of ipsilateral stroke and is an independent predictor, similar to stenosis	2B
Juxtaluminal hypoechoic area in ultrasonic images of carotid plaques and hemispheric symptoms	Griffin et al.,^8^ 2010	To assess the diagnostic value of presence of a juxtaluminal hypoechoic area without a visible echogenic cap.	A predictive model based on these parameters identified 77% of high-risk plaques associated with symptoms, with an OR of 6.7 and p < 0.001.	2
Dynamic carotid plaque imaging using ultrasonography	Giannopoulos et al.,^9^ 2020	To determine the prevalence of discordant motion in symptomatic and asymptomatic carotid plaques, develop a measure of its severity, and determine its correlation with prevalence of symptoms.	Discordant motion is more common in symptomatic cases than in asymptomatic cases.	2B
Indicações para ecodoppler de carótidas em pacientes assintomáticos – estamos solicitando corretamente?	Silva et al.,^10^ 2023	To determine whether specialists order carotid Doppler ultrasonography based on scientific evidence, using the parameters indications for examination, presence of risk factors, and medical specialty of physician.	A total of 36% of requests were appropriate. Hypertension was the most common risk factor and vascular surgeons were most likely to order scans correctly, with an OR of 3.52 and p = 0.02	3
Analysis of atherosclerotic plaque distribution in the carotid artery	Yang et al.,^11^ 2022	To determine whether the most common site of atherosclerotic plaque formation in the carotid arteries varies between initial and late stages.	Atherosclerotic plaques are most common in the anterior and posterior sections of the carotid arteries in cases of mild stenosis and are less frequent in lateral areas.	2B
Time-Resolved Wall Shear Rate Mapping Using High-Frame-Rate Ultrasound Imaging	Chee et al.,^12^ 2022	To develop WASHI, a noninvasive technique for mapping wall shear rate in carotid arteries and improve diagnosis of atherosclerosis.	WASHI was tested with *in vitro* models of the carotid bifurcation, showing high precision (mean error of 4.6%) and the capacity to track arterial wall motion, showing oscillating shear patterns and high WSR in the diseased model.	5
Wall shear rate and energy loss coefficient measures using conventional Doppler ultrasound do not predict carotid plaque progression.	Goudot et al.,^13^ 2023	The objective was to determine whether WSR and ELC could predict progression of carotid atherosclerosis using Doppler ultrasound.	Measurements of WSR and ELC obtained using conventional Doppler ultrasound did not exhibit a significant correlation with morphology or progression of carotid plaques.	2B
The Paradox Effect of Calcification in Carotid Atherosclerosis: Microcalcification is Correlated with Plaque Instability	Montanaro et al.,^14^ 2021	To investigate the association between histopathological characteristics, calcifications, inflammatory biomarkers, and risk factors in carotid plaques.	Microcalcifications were associated with highly inflamed and unstable plaques, whereas macrocalcifications appeared to stabilize them and were associated with M2 macrophage polarization.	3B
Criteria for Carotid Atherosclerotic Plaque Instability.	Ignatyev et al.,^15^ 2021	To determine the criteria for carotid plaque instability using duplex ultrasonography, elastography, histological analysis, fluorescent immunoassay, and electron paramagnetic resonance, correlating with histological findings.	Plaques with area > 90 mm^2^, plaque volume index > 0.6 cm^3^, and juxtaluminal black area ≥ 8 mm^2^ were robust predictors of instability, with a 94% prevalence of unstable plaques with these indicators. Immunohistochemical analysis revealed increased inflammatory cells and neovascularization in unstable plaques.	2B
Carotid plaque imaging and the risk of atherosclerotic cardiovascular disease	Zhu et al.,^16^ 2020	To review studies of imaging methods such as computed tomography, magnetic resonance, ultrasonography, and intravascular ultrasonography for analysis of features of carotid plaque vulnerability and their correlation with cardiovascular risk.	Techniques such as computed tomography and magnetic resonance are superior for detection of carotid plaque vulnerability.	5
Carotid Plaque Neovascularization Detected With Superb Microvascular Imaging Ultrasound Without Using Contrast Media	Zamani et al.,^17^ 2019	Assess the feasibility of using ultrasound with microvascular imaging to detect intraplaque neovascularization in carotid plaques, comparing it with conventional ultrasound, ultrasound with contrast, and histological assessments.	Superb microvascular imaging showed a good correlation with contrast-enhanced ultrasound and identified higher numbers of neovessels in plaques. This is an effective noninvasive option for assessing the stability of carotid plaques.	2B
External carotid artery plaques are associated with intracranial stenosis in patients with advanced coronary artery disease	Valaikiene et al.,^18^ 2019	To assess the association between plaques in the external carotid artery and asymptomatic intracranial stenosis in patients with advanced coronary artery disease diagnosed by extracranial duplex ultrasonography and color transcranial ultrasonography.	Plaques were found in the external carotid artery in 62.7% of the patients and were associated with a higher incidence of intracranial stenosis. Regression models showed that presence of plaques in the external carotid artery and significant stenosis in the internal carotid artery are important factors in intracranial stenosis.	3B
Increased serum chemerin levels associated with carotid intima-media thickness	Demi̇r et al.,^19^ 2021	To analyze the association between serum chemerin levels and carotid artery wall thickness as a marker of atherosclerosis.	Elevated serum chemerin levels are associated with carotid artery wall thickness, suggesting a link with cerebrovascular atherosclerotic disease.	2B
Early identification of carotid vulnerable plaque in asymptomatic patients	Jiao et al.,^20^ 2020	To associate identification of vulnerable carotid plaques with color Doppler ultrasonography and serum markers (MMP-9, LOX-1, and YKL-40), using statistical tests and logistic regression to analyze the data.	High MMP-9, LOX-1, and YKL-40 levels are effective indicators for detecting unstable plaques, with accuracy of 84.92%. This combination of tests is superior to traditional methods for identification of vulnerable plaques in asymptomatic patients.	2B
An Ultrasonographic Multiparametric Carotid Plaque Risk Index Associated with Cerebrovascular Symptomatology: A Study Comparing Color Doppler Imaging and Contrast-Enhanced Ultrasonography.	Rafailidis et al.,^21^ 2019	To compare ultrasonographic multiparametric indices using color Doppler imaging and contrast-enhanced sonography to assess symptomatic and asymptomatic carotid plaques.	Symptomatic plaques had greater stenosis, lower gray-scale median, and higher vulnerability indices. Contrast-enhanced ultrasonography had the best diagnostic precision, but without statistical significance compared with the other methods.	3A
Cerebral Ischemic Events Ipsilateral to Carotid Artery Stenosis. The Carotid Asymptomatic Stenosis (CARAS) Observational Study: First Year Preliminary Results.	Pini et al.,^22^ 2022	To estimate the risk of cerebral ischemic events in patients with asymptomatic carotid stenosis under medical treatment during the first year of follow-up.	In the first year, 2.3% of the patients had ipsilateral ischemic events. Plaque progression was observed in 14% of the patients and was associated with more neurological events. There were six deaths (2%) during the period.	2B
Five Year Outcomes in Men Screened for Carotid Artery Stenosis at 65 Years of Age: A Population Based Cohort Study	Högberg et al.,^23^ 2019	To analyze outcomes in 65-year-old men after 5 years of carotid ultrasound monitoring and identify factors contributing to disease progression.	Among men reassessed at 70 years of age, 1% with normal carotids and 3.6% with plaques progressed to moderate stenosis. Progression to severe stenosis was rare, with symptoms in 0.6%. Disease regression was common. Factors such as smoking and hypercholesterolemia were associated with progression.	1B
Cost-effectiveness of Assessing Ultrasound Plaque Characteristics to Risk Stratify Asymptomatic Patients With Carotid Stenosis.	Baradaran et al.,^24^ 2019	To analyze the cost-effectiveness of using ultrasound to identify high-risk carotid plaques in asymptomatic patients and to determine who required surgery.	Revascularization based on plaque echolucency was more cost-effective, with a lower cost per additional quality‐adjusted life year compared to strategies based on progression of stenosis only.	2C
Accuracy of duplex ultrasonography vs. angiotomography for the diagnosis of extracranial internal carotid stenosis.	Daolio et al.,^25^ 2024	To assess the precision, sensitivity, and specificity of duplex ultrasonography compared with angiotomography for detection of stenosis of the internal carotid artery, with 45 patients and 84 arteries analyzed.	Duplex ultrasound had accuracy of 69% for 50-94% stenosis and 84% for 70-94% stenosis. Analysis with angiotomography exhibited significant variations between evaluators, affecting clinical conduct in more than 37% of cases.	1B

OR = odds ratio; WSR = wall shear rate; ELC = energy loss coefficient.

## RESULTS

The PubMed search using the medical subject headings in English (Echocardiography, Doppler OR Doppler Ultrasonography) AND (Carotid Artery Diseases OR Arterial Diseases OR Carotid Artery Atherosclerosis) AND (Atheroma OR Atherosclerotic Plaque) AND (Diagnostic Evaluation OR Diagnostic Imaging OR Ultrasonographic Evaluation) returned a total of 631 articles. Of these, 69 met the preestablished inclusion criteria: full text articles published from 2018 to 2024. Sixteen of those 69 articles presented detailed analyses of atherosclerotic plaques using Doppler ultrasonography, exploring both their genesis and their final outcomes, all of which used the same imaging process.

Searching the SciELO database identified 22 articles, 11 of which were within the selected period. Three of these were selected for the integrative review. In turn, the BVS database did not return any articles when searched with the same Boolean operators used for the other databases, indicating that there were no items that corresponded with the search criteria used. Finally, application of the inclusion and exclusion criteria and the Boolean operators to the Cochrane database returned just seven articles, none of which were chosen for the review because of their content and methodological aspects.

## DISCUSSION

The decision to intervene in carotid disease is often taken on the basis of the degree of stenosis observed in the common carotid artery and its branches and also on the basis of recent symptoms. However, a considerable proportion of patients have asymptomatic atherosclerotic plaques in their carotid arteries, suggesting a high risk of stroke in the future, compared with people who have had symptomology recently. As imaging methods have improved and become increasingly available and utilized in physicians’ offices, increasing effort has been expended to characterize these risks, taking account not only of the degree of occlusion caused by plaques, but also their morphological characteristics. This is achieved using imaging methods such as computed tomography, magnetic resonance, and/or Doppler ultrasonography.^7,8^

Of all of the different imaging methods employed, Doppler ultrasonography remains the techniques most widely used to assess carotid disease. This is because it yields information on the degree of stenosis and plaque morphology, enabling their content to be determined, such as presence or absence of calcifications, the quality of the fibrous cap, and the degree of stability of the plaque. It is also a rapid, accessible, and noninvasive method.^9,10^

Duplex scanning, also known as Doppler ultrasonography, can be used to evaluate the degree of arterial stenosis and narrowing and assess the surface of the lumen and the extent of involvement of the artery. This is achieved by evaluating intima‐media thickness and the echotextures present in the plaque adhering to the carotid. Additionally, it is also possible to estimate the risk of stroke by Doppler‐derived velocity.^11^

Formation of initial stage atherosclerotic plaques predominantly occurs at the external wall of the carotid bulb and the anterior and posterior portions of the common carotid artery. These areas have decreased shear stress and stasis of blood flow and stress, which together contribute to flow reversal.^11-13^ Over time, these plaques can increase in size, attaining different degrees of stenosis, and are influenced by factors that can affect their stability.^14^

It is known that atherosclerotic plaques can be stable or unstable. When unstable, they are susceptible to erosion, fissures, or ruptures, which provoke leakage of the lipid core, intraplaque hemorrhage, inflammation, and accentuated neovascularization, contributing to thrombosis, occlusion, and acute myocardial infarction, which precede stenotic processes considered hemodynamically significant.^15-17^ It is important to point out that the likelihood of rupture increases further still when calcifications are present and that, despite the widespread adoption of Doppler ultrasonography as the preferred technique for assessing carotid stenosis, analysis of lesions can be compromised by formation of acoustic shadows generated by already calcified atherosclerotic plaques, making it more difficult to quantify the degree of vessel stenosis.^14,18^

It is important to assess atherosclerotic plaques in the carotid arteries to estimate the risk of ischemic events, such as stroke. Yang et al.^11^ used duplex scanning to analyze plaque morphology and hemodynamics, correlating the findings with risk of stroke. During the examination, which was conducted with patients in the supine position with the head in contralateral rotation, axial and longitudinal images of the common and internal carotid arteries revealed characteristics such as hypoechoic areas and juxtaluminal black areas, indicative of lipid rich plaques and elevated inflammatory activity. These areas, which have low echogenicity on Doppler ultrasonography, were associated with a greater risk of rupture and formation of thrombi and the juxtaluminal black area were also related to a fibrous cap and lipid-rich core.^11^ Rapid progression of these plaques, characterized by increased volume and length, was identified as an additional risk factor for obstruction of the arterial lumen and for embolic events. These findings emphasize the correlation between morphological characteristics of the plaques and the risk of stroke, highlighting the role of duplex scanning in risk stratification and planning of guided therapeutic interventions.^11,16^

Using the same imaging method, studies have demonstrated that plaques with areas > 90 mm^2^ (odds ratio [OR] = 4.05; 95%CI 1.32-13; p = 0.006), volume index > 0.6cm^3^ (95%CI, 1.05-9.58; p = 0.04), and juxtaluminal black areas ≥ 8 mm^2^ (OR = 2.82; 95%CI 1.22-6.23; p = 0.02) function as predictors indicative of histological plaque instability. This fact is corroborated by the observation that 94 in every 100 patients with these indicators had unstable plaques, in addition to significant increases in inflammatory cells found in the inflammatory process, which occurs between the atherosclerotic plaque cap and the endothelium.^15^

Some characteristics of atherosclerotic plaques detected by arterial Doppler ultrasonography, such as carotid intima-media thickness (CIMT), are significant markers of subclinical atherosclerosis. Patients with ischemic stroke exhibited significantly higher CIMT compared with healthy controls (p < 0.001). Additionally, elevated serum levels of chemerin, an inflammatory adipokine, were positively correlated with CIMT (p < 0.05), indicating a relationship between chronic inflammation, increased arterial thickness, and a greater degree of atherosclerosis. These findings emphasize the role that Doppler ultrasonography has to play in early detection of atherosclerotic plaques, which are precursors of ischemic events. Instability of these plaques, which is associated with increased CIMT and exacerbated inflammation, can lead to rupture, formation of thrombi, and cerebral ischemia. As such, arterial Doppler ultrasonography is essential, not only to diagnose atherosclerosis, but also to stratify the risk of ischemic stroke and guide more effective preventative interventions.^19^

Moreover, arterial Doppler ultrasonography can be supplemented with use of serum markers for analysis of plaque stability. The combination of duplex scanning with markers such as matrix metalloproteinase 9 (MMP-9), lectin-like oxidized low-density lipoprotein receptor-1 (LOX-1), and chitinase-3-like protein (YKL-40) is capable of early identification of vulnerable plaques in the carotid arteries in asymptomatic patients, achieving sensitivity of 87.67%, specificity of 81.13%, and diagnostic accuracy of 84.92%. It is thus possible to identify groups with elevated risk for cardiovascular and cerebrovascular events.^16,20^

The markers MMP-9, LOX-1, and YKL-40 are widely studied in the literature as indicators of atherosclerotic plaque instability. At elevated levels, they have been consistently associated with increased risk of plaque rupture. Each of these markers represents specific biological processes that contribute to plaque vulnerability. MMP-9 participates in degradation of the extracellular matrix, weakening the fibrous cap and enabling infiltration of inflammatory cells, which increases the probability of rupture. In turn, LOX-1 provokes endothelial dysfunction by internalizing oxidated lipoproteins and triggering inflammatory and apoptotic responses and is related to hypoechoic and isoechoic plaques that are detectable by duplex scan, which are characteristic of structural vulnerability. YKL-40 (chitinase-3-like protein) is an inflammatory protein involved in tissue remodeling. High levels indicate a chronic inflammatory state, which is instrumental in destabilization of plaques and atherosclerotic progression. Since these markers indicate structural plaque vulnerability, they enable a more targeted approach to risk stratification and therapeutic interventions.^18^

Generally, carotid arteries with symptomatic plaques and a significant degree of occlusion exhibit a lower grey scale and a higher Kanbner index, which is the result of a combination of the degree of stenosis, median grey scale, and a quantitative measure of surface irregularities, obtained by means of colored Doppler imaging and contrast enhanced ultrasonography. These factors are essential for analysis of plaque vulnerability during Doppler ultrasonography.^21^ This method enables image analysis to reveal possible unfavorable situations since, as carotid disease progresses, patients become more susceptible to outcomes such as ipsilateral ischemic stroke and transitory ischemic attacks.^19^ During the analysis conducted with Doppler ultrasonography, it is observed that as stenosis progresses the risk of development of unfavorable outcomes correlated with neurological events increases (p = 0.001, OR = 8.9; 95%CI 1.9-4,1).^22,23^

Doppler ultrasonography can be used to analyze movement of the carotid plaque. In dynamic analyses, imaging studies demonstrate that during systole and the start of diastole, all of the components present in the carotid plaque may move in the same direction, generating concordant motion in some of these plaques. However, in other cases, the plaque may move in different directions, creating what is known as discordant motion. Discordant motion is indicative of presence of elevated stress on the plaque, which, when quantified, may serve as an indirect measure of the severity of the risk of rupture and, logically, the risk of development of symptoms. The prevalence of discordant motion was 89.1% in symptomatic plaques and 17.9% in asymptomatic plaques.^9^

Considering the multifaceted characteristics that atherosclerotic carotid plaques can exhibit on ultrasound, the importance for appropriate therapeutic decision making of the multiparametric assessment discussed above is clear.^21^ Evaluation of the risks of undesirable events, the costs involved, and the possibility of better quality of life are crucial elements to be considered. These aspects underscore the advantages of Doppler ultrasound in terms of its cost-benefit ratio for assessing atherosclerotic lesions, thereby contributing to identification of patients with symptomatic and asymptomatic stenosis and supporting decision-making on the need for carotid endarterectomy, where applicable.^24^

Assessment of atherosclerotic plaques in the carotid arteries is essential to prevent risks of cardiovascular diseases. While use of Doppler ultrasonography is widespread because of its accessibility and capacity to offer information in real time, its use in isolation may limit detection of the diversity of plaque characteristics. Studies show that supplementary methods such as magnetic resonance and computed tomography are more sensitive for identification of components such as intraplaque hemorrhage, lipid-rich necrotic core, and calcifications, all of which are important predictors of cardiovascular events.^25^ Moreover, advanced techniques such as positron emission tomography enable visualization of inflammatory processes, extending comprehension of the pathophysiology of plaques. Integration of these methods offers a more precise morphological assessment, validated by correlation with histological findings, supporting more targeted and effective clinical interventions.^15,24^

While the present review presents a comprehensive analysis of Doppler ultrasonography for assessment of atherosclerotic plaques, it is exclusively focused on investigation of carotid disease using this method and does not enter into in-depth comparisons with other diagnostic techniques, such as angiotomography or magnetic resonance. Moreover, the present study is limited to reviewing literature published from 2018 to 2024, which restricts the scope of the analysis.

This temporal restriction was adopted with the objective of prioritizing recent evidence, reflecting the most up-to-date developments in clinical practice, technologies, and therapeutic management in the area. It was considered that the last 7 years. constitute a sufficient time span to capture contemporary tendencies in the scientific literature, aligned with the most recent guidelines. Notwithstanding, it is recognized that this choice could have excluded classic studies or trials with considerable methodological rigor published prior to this period, constituting a limitation of the review.

While the present integrative review analyzed a considerable number of studies with a certain methodological and thematic homogeneity, a meta-analysis was not conducted because of the variability of the outcomes analyzed and differences in the diagnostic criteria, populations studied, and statistical methods employed. This heterogeneity would have compromised both the statistical and the clinical validity of a combined quantitative analysis. In view of this, a critical synthesis was conducted, which is more appropriate to the profile of the available data.

This study makes a relevant contribution to vascular surgeons’ practice, emphasizing the role of Doppler ultrasound as a diagnostic tool for characterization of carotid atherosclerotic plaques. In contrast to the guidelines of the Brazilian Society of Angiology and Vascular Surgery and those of European societies, which predominantly focus on the degree of carotid stenosis as the principal criterion for indicating intervention, this article extends the approach by highlighting the importance of multiparametric analysis of atherosclerotic plaques, considering morphological characteristics such as echogenicity and calcifications and also serum biomarkers (MMP-9, LOX-1, and YKL-40) to conduct a more precise risk stratification. This perspective suggests a possible evolution of future recommendations, incorporating additional criteria, in addition to percentage stenosis, to define therapeutic management in a more personalized manner.^26^

## CONCLUSIONS

Doppler ultrasonography plays an essential role in assessment of atherosclerotic plaques in carotid arteries, enabling noninvasive analysis of their morphology and estimating the risk of cerebrovascular events such as stroke. This study’s detailed analysis highlights specific plaque attributes, emphasizing the value of Doppler ultrasound for risk stratification and clinical decision making. Additionally, it also contributes significantly by emphasizing the relevance of duplex scanning as a cost-effective and practical method, integrating use of serum markers and multiparametric analyses. Based on the established literature, the study has highlighted the importance of Doppler as a cost-effective and practical tool, supporting clinical decision making and enriching strategies for risk stratification in medical practice.

## Data Availability

Dados incluídos no artigo/material suplementar: “Todos os dados gerados ou analisados estão incluídos neste artigo e/ou no material suplementar”.
