# Supplementary material for: Attributes of atherosclerotic plaques in carotid artery disease: a Doppler ultrasound assessment
Source: J Vasc Bras. 2025 Oct 27;24:e20240186. doi: 10.1590/1677-5449.202401862 (PMC12642919; doi:10.1590/1677-5449.202401862)
Supplement: Translation

# Translation: Atributos da placa de ateroma na doença carotídea: uma avaliação com ultrassonografia Doppler

**DOI:** 10.1590/1677-5449.202401861

**Authors:** Dyana Carolina Teixeira Trevisan, Carla Aparecida Faccio Bosnardo

**Affiliations:** 1 São Leopoldo Mandic de Araras – SLMA, Araras, SP, Brasil.

**Keywords:** ultrassonografia Doppler, doença carotídea, placa de ateroma

## Abstract

A aterosclerose, uma inflamação crônica da parede arterial, resulta de lesões no endotélio vascular e é influenciada por diversos fatores de risco. A estenose carotídea, marcador crucial de risco cardiovascular, é especialmente relevante em homens acima de 65 anos de idade. O tratamento e métodos diagnósticos da doença carotídea são temas de debate. Nesta revisão integrativa, foca-se na avaliação da placa aterosclerótica por ultrassonografia Doppler. A busca nas bases de dados PubMed e SciELO resultou em 69 artigos, dos quais 16 detalharam essa análise. O ultrassom Doppler emergiu como a modalidade mais utilizada devido à sua não invasividade e à sua capacidade de fornecer informações detalhadas sobre a morfologia da placa e o fluxo sanguíneo. Discute-se que o eco-Doppler permite uma avaliação abrangente da placa, identificando características morfológicas e estimando o risco de complicações. Conclui-se que a ultrassonografia Doppler é essencial na avaliação da doença carotídea, fornecendo uma abordagem não invasiva e detalhada, contribuindo para a identificação precoce de pacientes em alto risco e melhorando os resultados clínicos.

## INTRODUÇÃO

A aterosclerose é uma condição caracterizada pela inflamação crônica e reparativa da parede arterial em resposta a lesões no endotélio vascular, sendo influenciada por múltiplos fatores de risco, como genética, idade, sexo, hiperlipidemia, hipertensão arterial e tabagismo^1^. Em condições normais, o endotélio arterial desempenha um papel significativo na proteção do vaso sanguíneo. Contudo, em resposta a condições traumáticas, pode ocorrer lesão, promovendo um processo patológico que resulta no acúmulo de lipídios envoltos por cápsula fibrosa aderida à parede da artéria, dando origem à placa aterosclerótica. Com a progressão da doença, a placa pode passar por processo de calcificação^1,2^.

Essas placas ateroscleróticas podem apresentar risco de ruptura, favorecendo a formação de trombos que podem se impactar e ocluir outros vasos sanguíneos, levando a potenciais complicações, como acidente vascular cerebral (AVC), caso acometam as carótidas. A depender do local de impactação do trombo, esse AVC pode se manifestar como isquêmico, especialmente quando a origem do trombo está em placas instáveis da carótida interna, que irriga diretamente o cérebro^2,3^.

A estenose carotídea é um marcador importante de risco cardiovascular, estando associada a uma prevalência variável na população e contribuindo significativamente para casos de isquemia cerebral. Essa condição tende a afetar mais homens do que mulheres, especialmente aqueles com mais de 65 anos de idade^2-5^.

O tratamento da doença aterosclerótica carotídea é objeto de discussão contínua devido à sua importância clínica, com constantes avanços teóricos e tecnológicos. Além das opções terapêuticas, há debates sobre os métodos de diagnóstico e sua influência nas decisões médicas. Em casos de estenose carotídea, a angiotomografia computadorizada e o ultrassom Doppler são opções comuns para a avaliação da placa^4^.

Embora forneça informações detalhadas, a angiotomografia computadorizada é um procedimento invasivo, caro e envolve o uso de contraste iodado, sendo contraindicado para alguns pacientes. Por outro lado, o ultrassom Doppler é não invasivo, acessível e seguro, oferecendo informações importantes sobre a anatomia e o fluxo sanguíneo sem exposição à radiação ionizante ou ao contraste, sendo adequado para gestantes e pacientes alérgicos, por exemplo^6^.

Diante do exposto, este artigo propõe uma revisão integrativa com o intuito de salientar as vantagens do emprego do ultrassom Doppler na avaliação da placa aterosclerótica. Seu objetivo primordial é fornecer subsídios que contribuam para a análise e qualificação das características da placa de ateroma identificadas por meio da ultrassonografia Doppler, evidenciando como essa abordagem pode ser eficaz na tomada de decisões clínicas e na definição da conduta terapêutica.

## MÉTODOS

Foi realizada uma revisão integrativa da literatura, com foco na caracterização das placas de ateroma em doenças carotídeas, por meio da avaliação com ultrassonografia Doppler. O estudo foi norteado pela seguinte questão: “Quais são as características das placas de ateroma detectadas pelo eco-Doppler arterial em pacientes com doença carotídea, e como essas características influenciam o risco de ruptura, isquemia e desenvolvimento de AVC?”

Para a resolução do questionamento, foi utilizada a estratégia PICO (paciente/intervenção/controle/desfecho [*outcome*]). As buscas foram realizadas nas bases de dados PubMed, Cochrane Library, Biblioteca Virtual em Saúde (BVS) e SciELO, utilizando descritores em Ciências da Saúde (DeCs). Os termos de busca foram combinados com os operadores booleanos AND e OR para maximizar a sensibilidade, conforme segue: (Echocardiography, Doppler OR Doppler Ultrasonography) AND (Carotid Artery Diseases OR Arterial Diseases OR Carotid Artery Atherosclerosis) AND (Atheroma OR Atherosclerotic Plaque) AND (Diagnostic Evaluation OR Diagnostic Imaging OR Ultrasonographic Evaluation).

A busca também foi realizada em português na SciELO, utilizando a seguinte estratégia: ((ultrassonografia Doppler OR duplex scan) AND (carótidas OR carótida OR doenças arteriais OR aterosclerose das carótidas)) AND ((placa de ateroma OR placa aterosclerótica) AND (avaliação diagnóstica OR diagnóstico por imagem OR avaliação ultrassonográfica)).

Foram consideradas para inclusão na revisão publicações entre os anos de 2018 e 2024, escritas em português ou inglês, que abordassem especificamente o eco-Doppler vascular de carótida e suas associações com aterosclerose. Artigos incompletos, teses, dissertações e estudos que não estivessem diretamente relacionados à pergunta norteadora foram excluídos.

Para a sistematização dos dados, foi elaborada uma tabela padronizada contendo as seguintes informações: autores, título do artigo, ano de publicação, tipo de estudo, objetivos, parâmetros de análise e principais resultados, bem como análise segundo os níveis de evidência de Oxford. A tabela foi organizada em ordem de citação durante a discussão do presente artigo. A análise crítica dos artigos selecionados, realizada a partir de uma leitura minuciosa, permitiu discutir os achados e responder à questão norteadora do estudo.

Dois revisores conduziram de forma independente a seleção dos estudos, com base na leitura de títulos e resumos. Os artigos selecionados foram, então, submetidos à leitura completa e discussão. Em caso de divergência de opiniões, um terceiro revisor foi consultado para tomar uma decisão final.

Este projeto foi dispensado de aprovação pelo Comitê de Ética em Pesquisa, uma vez que utilizou exclusivamente dados disponíveis publicamente em bases de dados, não envolvendo a participação direta de humanos.

Todos os dados foram organizados de acordo com a Tabela 1, onde constam todos os estudos clínicos selecionados e utilizados para compor a presente revisão integrativa de literatura e sustentar a importância do estudo das placas ateroscleróticas através do eco-Doppler arterial para prevenir eventos isquêmicos, como AVC.

**Tabela 1:** Sumário de estudos clínicos e revisões selecionadas utilizadas para compor a presente revisão integrativa de literatura e sustentar a importância do estudo das placas ateroscleróticas através do eco-Doppler arterial para prevenir eventos isquêmicos como AVC.

**Título**	**Autor e ano de publicação**	**Objetivos e parâmetros de análise**	**Resultados**	**Nível de evidência de Oxford**
Prediction of Stroke Risk by Detection of Hemorrhage in Carotid Plaques: Meta-Analysis of Individual Patient Data	Schindler et al.^7^, 2020	Estudo de coorte, com 560 pacientes, que avaliou o risco de AVC em estenose carotídea, considerando a presença de hemorragia intraplaca na ressonância magnética.	Hemorragia intraplaca, mais comum em estenose carotídea assintomática, aumenta o risco de AVC ipsilateral e é preditor independente, assim como a estenose.	2B
Juxtaluminal hypoechoic area in ultrasonic images of carotid plaques and hemispheric symptoms	Griffin et al.^8^, 2010	Avaliar o valor diagnóstico da presença de uma área hipoecogênica justaluminal sem capa ecogênica visível.	O modelo preditivo baseado nesses parâmetros identificou 77% das placas de alto risco associadas a sintomas, com OR de 6,7 e p < 0,001.	2
Dynamic carotid plaque imaging using ultrasonography	Giannopoulos et al.^9^, 2020	Determinar a prevalência do movimento discordante em placas carotídeas sintomáticas e assintomáticas, desenvolver uma medida de sua gravidade e avaliar a correlação com a prevalência de sintomas.	O movimento discordante é mais frequente em casos sintomáticos do que em assintomáticos.	2B
Indicações para ecodoppler de carótidas em pacientes assintomáticos – estamos solicitando corretamente?	Silva et al.^10^, 2023	Avaliar se os especialistas solicitam eco-Doppler de carótidas com base em evidências cientificas, utilizando como parâmetros a indicação do exame, presença de fatores de risco e especialidade médica solicitante.	Um total de 36% das solicitações foram adequadas, a hipertensão foi o fator de risco mais comum, e os cirurgiões vasculares foram os mais assertivos na escolha, com OR de 3,52 e p = 0,02	3
Analysis of atherosclerotic plaque distribution in the carotid artery	Yang et al.^11^, 2022	Determinar que a região mais comum de se formar as placas de ateroma nas artérias carótidas varia entre os estágios iniciais e tardios.	Placas ateroscleróticas são mais comuns nas seções anterior e posterior das carótidas em casos de estenose leve, sendo menos frequentes nas seções laterais.	2B
Time-Resolved Wall Shear Rate Mapping Using High-Frame-Rate Ultrasound Imaging	Chee et al.^12^, 2022	Desenvolver o WASHI, uma técnica não invasiva para mapear a taxa de cisalhamento da parede nas artérias carotídeas e aprimorar o diagnóstico da aterosclerose.	O WASHI foi testado em modelos *in vitro* e de bifurcação carotídea, mostrando alta precisão (erro médio de 4,6%) e capacidade de rastrear o movimento da parede arterial, com padrões de cisalhamento oscilantes e alta WSR em modelos doentes.	5
Wall shear rate and energy loss coefficient measures using conventional Doppler ultrasound do not predict carotid plaque progression.	Goudot et al.^13^, 2023	O objetivo foi avaliar se a WSR) e o ELC podem prever a progressão da aterosclerose carotídea usando ultrassom Doppler.	As medidas de WSR e ELC obtidas por ultrassom Doppler convencional não apresentaram correlação significativa com a morfologia ou progressão das placas carotídeas.	2B
The Paradox Effect of Calcification in Carotid Atherosclerosis: Microcalcification is Correlated with Plaque Instability	Montanaro et al.^14^, 2021	Investigar a associação entre características histopatológicas, calcificações, biomarcadores inflamatórios e fatores de risco em placas carotídeas mini.	Microcalcificações foram associadas a placas altamente inflamadas e instáveis, enquanto macrocalcificações parecem estabilizá-las, estando ligadas à polarização de macrófagos M2.	3B
Criteria for Carotid Atherosclerotic Plaque Instability.	Ignatyev et al.^15^, 2021	Determinar os critérios de instabilidade da placa carotídea por meio de ultrassonografia *duplex*, elastografia, análise histológica, imunoensaio fluorescente e ressonância paramagnética eletrônica, correlacionando com achados histológicos.	Plaquetas com área > 90 mm^2^, índice de volume > 0,6 cm^3^ e área justaluminal preta ≥ 8 mm^2^ foram fortes preditores de instabilidade, com 94% de prevalência de placas instáveis nessas condições. A análise imunohistoquímica encontrou mais células inflamatórias e de neovascularização em placas instáveis.	2B
Carotid plaque imaging and the risk of atherosclerotic cardiovascular disease	Zhu et al.^16^, 2020	Revisar estudos sobre métodos de imagem, como tomografia computadorizada, ressonância magnética, ultrassonografia e ultrassonografia intravascular, para analisar as características vulneráveis da placa carotídea e sua correlação com o risco cardiovascular.	Técnicas como tomografia computadorizada e ressonância magnética são superiores em detectar vulnerabilidade de placa carotídea.	5
Carotid Plaque Neovascularization Detected With Superb Microvascular Imaging Ultrasound Without Using Contrast Media	Zamani et al.^17^, 2019	Avaliar a viabilidade do ultrassom com imagem microvascular para detectar neovascularização intraplaca em placas carotídeas, comparando-o com ultrassom convencional, ultrassom com contraste e avaliações histológicas.	A imagem microvascular superior mostrou boa correlação com a ultrassonografia com realce por contraste e identificou mais neovesséis em placas. É uma alternativa não invasiva eficaz para avaliar a estabilidade das placas carotídeas.	2B
External carotid artery plaques are associated with intracranial stenosis in patients with advanced coronary artery disease	Valaikiene et al.^18^, 2019	Avaliar a associação entre placas na artéria carótida externa e estenose intracraniana assintomática em pacientes com doença arterial coronariana avançada, diagnosticada por ultrassonografia *duplex* extracraniana e ultrassonografia transcraniana colorida.	Placas na artéria carótida externa foram encontradas em 62,7% dos pacientes e associadas a uma maior incidência de estenose intracraniana. Modelos de regressão mostraram que a presença de placas na artéria carótida externa e estenose significativa na artéria carótida interna são fatores importantes para a estenose intracraniana.	3B
Increased serum chemerin levels associated with carotid intima-media thickness	Demi̇r et al.^19^, 2021	Analisar a associação entre os níveis séricos de quemerina e a espessura da parede arterial carótida como marcador de aterosclerose.	Níveis elevados de quemerina sérica estão associados à espessura da parede arterial carótida, sugerindo uma ligação com a doença cerebrovascular aterosclerótica.	2B
Early identification of carotid vulnerable plaque in asymptomatic patients	Jiao et al.^20^, 2020	Associar a identificação de placas vulneráveis carotídeas com ultrassonografia Doppler colorida e marcadores séricos (MMP-9, LOX-1 e YKL-40), utilizando testes estatísticos e regressão logística para análise dos dados.	Altos níveis de MMP-9, LOX-1 e YKL-40 são indicadores eficazes para detectar placas instáveis, com uma acurácia de 84,92%. Essa combinação de testes se mostra superior aos métodos tradicionais na identificação de placas vulneráveis em pacientes assintomáticos.	2B
An Ultrasonographic Multiparametric Carotid Plaque Risk Index Associated with Cerebrovascular Symptomatology: A Study Comparing Color Doppler Imaging and Contrast-Enhanced Ultrasonography.	Rafailidis et al.^21^, 2019	Comparar índices multiparamétricos ultrassonográficos, utilizando Doppler colorido e ultrassonografia com contraste para avaliar placas carotídeas sintomáticas e assintomáticas.	Placas sintomáticas apresentaram maior estenose, menor mediana de escala de cinza e índices de vulnerabilidade mais altos. A ultrassonografia com contraste demonstrou a melhor precisão diagnóstica, embora sem significância estatística em comparação com os outros métodos.	3A
Cerebral Ischemic Events Ipsilateral to Carotid Artery Stenosis. The Carotid Asymptomatic Stenosis (CARAS) Observational Study: First Year Preliminary Results.	Pini et al.^22^, 2022	Estimar o risco de eventos isquêmicos cerebrais em pacientes com estenose carotídea assintomática em tratamento médico durante o primeiro ano de acompanhamento.	No primeiro ano, 2,3% dos pacientes tiveram eventos isquêmicos ipsilaterais. A progressão das placas foi encontrada em 14% dos pacientes e associada a mais eventos neurológicos. Houve seis mortes (2%) no período.	2B
Five Year Outcomes in Men Screened for Carotid Artery Stenosis at 65 Years of Age: A Population Based Cohort Study	Högberg et al.^23^, 2019	Analisar os desfechos de homens de 65 anos após 5 anos de monitoramento por ultrassonografia de carótida e identificar os fatores que contribuem para a progressão da doença.	Entre os homens reavaliados aos 70 anos, 1% com carótidas normais e 3,6% com placas avançaram para estenose moderada. A progressão para estenose severa foi rara, com sintomas em 0,6%. Regressão da doença foi comum. Fatores como tabagismo e hipercolesterolemia foram associados à progressão.	1B
Cost Effectiveness of Assessing Ultrasound Plaque Characteristics to Risk Stratify Asymptomatic Patients With Carotid Stenosis.	Baradaran et al.^24^, 2019	Analisar a custo-efetividade de usar ultrassonografia para identificar placas carotídeas de alto risco em pacientes assintomáticos e determinar quem precisaria de cirurgia.	Revascularização guiada por ecogenicidade das placas foi mais custo-efetiva, com menor custo por ano de vida ajustado pela qualidade comparado a estratégias baseadas apenas na progressão da estenose.	2C
Accuracy of duplex ultrasonography versus angiotomography for the diagnosis of extracranial internal carotid stenosis.	Daolio et al.^25^, 2024	Avaliar a precisão, sensibilidade e especificidade da ultrassonografia duplex em comparação com a angiotomografia na detecção de estenose da artéria carótida interna, com 45 pacientes e 84 artérias analisadas.	O *duplex scan* teve uma precisão de 69% para estenoses de 50-94% e 84% para estenoses de 70-94%. A análise por angiotomografia apresentou variações significativas entre avaliadores, afetando o manejo clínico em mais de 37% dos casos.	1B

AVC = acidente vascular cerebral; OR = *odds ratio*; WSR = taxa de cisalhamento da parede (*wall shear rate*); ELC = coeficiente de perda de energia (*energy loss coeficient*).

## RESULTADOS

Utilizando os descritores de saúde em inglês (Echocardiography, Doppler OR Doppler Ultrasonography) AND (Carotid Artery Diseases OR Arterial Diseases OR Carotid Artery Atherosclerosis) AND (Atheroma OR Atherosclerotic Plaque) AND (Diagnostic Evaluation OR Diagnostic Imaging OR Ultrasonographic Evaluation), a busca no PubMed resultou em 631 artigos. Destes, 69 atenderam aos critérios de inclusão estabelecidos, sendo completos e tendo sido publicados no período de 2018 a 2024. Entre os 69 artigos, 16 forneceram uma análise detalhada da placa de ateroma por meio do eco-Doppler, explorando tanto a gênese quanto os desfechos finais, e todos utilizaram o mesmo processo de imagiologia.

Na base de dados SciELO, foram identificados 22 artigos, sendo que 11 estavam dentro do quinquênio selecionado. Destes, três foram selecionados para a elaboração da presente revisão integrativa. Por sua vez, na base de dados da BVS, nenhum artigo foi encontrado a partir do operador booleano utilizado nas demais, indicando que não houve correspondência com os critérios de busca empregados. Quanto à base de dados da Cochrane, a aplicação dos critérios de inclusão e exclusão, bem como o operador booleano, resultou em apenas sete artigos, dos quais nenhum foi considerado para compor a presente revisão, devido ao conteúdo e aspectos metodológicos.

## DISCUSSÃO

A intervenção para a doença carotídea é frequentemente determinada pelo grau de estenose presente na artéria carótida comum e seus ramos, bem como pela presença de sintomas recentes. No entanto, há uma considerável proporção de pacientes com placas ateroscleróticas assintomáticas em suas carótidas, sugerindo um alto risco de AVC no futuro, em comparação com indivíduos que apresentam sintomatologia recente. Com os avanços nos métodos de imagem e a sua crescente utilização nos consultórios médicos, tem-se observado um maior esforço para caracterizar esses riscos, levando em conta não apenas o grau de oclusão causado por essas placas, mas também suas características morfológicas. Isso tem sido realizado por meio de métodos de imagem como tomografia computadorizada, ressonância magnética e/ou ultrassonografia Doppler^7,8^.

Entre os vários métodos de imagiologia utilizados, a ultrassonografia Doppler continua sendo a modalidade mais utilizada para avaliar a doença carotídea. Isso se deve ao fato de que fornece informações sobre o grau de estenose e a morfologia da placa, permitindo a caracterização de seu conteúdo, como a presença ou ausência de calcificações, a qualidade da capa fibrótica e o nível de estabilidade da placa. Além disso, é um método rápido, acessível e não invasivo^9,10^.

O *duplex scan*, também conhecido como ultrassonografia Doppler, possibilita a avaliação do grau de estenose arterial, estreitamento e superfície do lúmen, bem como a extensão do envolvimento da artéria. Isso viabiliza uma avaliação da espessura médio-intimal e das ecotexturas presentes na placa aderida à carótida. Além disso, há a possibilidade de estimar o risco de AVC por meio da velocidade derivada do Doppler^11^.

A formação das placas ateroscleróticas em estágio inicial ocorre predominantemente na parede externa do bulbo carotídeo e nas porções anterior e posterior da artéria carótida comum. Essas áreas são caracterizadas pela redução da força de cisalhamento, estase de fluxo sanguíneo e estresse, que, quando somadas, contribuem para uma reversão do fluxo^11-13^. Ao longo do tempo, essas placas podem aumentar de tamanho e apresentar diferentes graus de estenose, sendo influenciadas por fatores que podem afetar sua estabilidade^14^.

Sabe-se que as placas ateroscleróticas podem ser estáveis ou instáveis. Quando instáveis, são suscetíveis a erosão, fissuras ou rupturas, que levam ao extravasamento de conteúdo lipídico, hemorragia intraplaca, inflamação e neovascularização acentuada, contribuindo para situações de trombose, oclusão e infarto agudo do miocárdio os quais precedem um processo estenótico considerado hemodinamicamente significativo^15-17^. É importante salientar que, quando há calcificações, as chances de ruptura se tornam ainda maiores e que, apesar da ampla adoção do eco-Doppler como técnica consagrada para avaliar a estenose carotídea, a análise da lesão pode ser comprometida pela formação de sombras acústicas geradas pelas placas ateroscleróticas já calcificadas, dificultando a quantificação do grau de estenose do vaso^14,18^.

A avaliação de placas ateroscleróticas nas artérias carótidas é importante para estimar o risco de eventos isquêmicos, como o AVC. Yang et al.^11^ utilizaram o *duplex scan* para analisar a morfologia das placas e a hemodinâmica, correlacionando os achados ao risco de AVC. Durante o estudo, realizado com pacientes em posição supina e cabeça em rotação contralateral, imagens axiais e longitudinais das artérias carótidas comuns e internas evidenciaram características como áreas hipoecoicas e regiões pretas justaluminais, indicativas de placas ricas em lipídios e elevada atividade inflamatória. Essas áreas, que apresentam baixa ecogenicidade na ultrassonografia Doppler, foram associadas a maior risco de ruptura e formação de trombos, sendo as regiões pretas justaluminais também relacionadas a uma capa fibrótica e um núcleo lipídico significativos^11^. A progressão rápida dessas placas, caracterizada pelo aumento de volume e extensão, foi identificada como fator adicional de risco para obstrução do lúmen arterial e eventos embólicos. Os achados reforçam a correlação entre características morfológicas das placas e o risco de AVC, destacando o papel do *duplex scan* na estratificação de risco e no planejamento de intervenções terapêuticas direcionadas^11,16^.

Utilizando o mesmo método de imagem, estudos demonstram que placas com áreas > 90 mm^2^ (*odds ratio* (OR) = 4,05; IC95% 1,32-13; p = 0,006), índice de volume > 0,6cm^3^ (IC95%, 1,05-9,58; p = 0,04) e área preta justaluminal ≥ 8 mm^2^ (OR = 2,82; IC95% 1,22-6,23; p = 0,02) servem como preditores para caracterização histológica de instabilidade de placa. Esse fato é corroborado pela observação de que 94 em cada 100 pacientes com esses indicadores apresentavam placas instáveis, além do aumento significativo de células inflamatórias comuns ao processo inflamatório, que ocorre entre a capa da placa aterosclerótica e o endotélio^15^.

As características das placas ateroscleróticas detectadas por ultrassonografia Doppler arterial, como a espessura médio-intimal da artéria carótida (*carotid intima-media thickness* [CIMT]), são marcadores significativos de aterosclerose subclínica. Pacientes com AVC isquêmico apresentaram CIMT significativamente maior em comparação a controles saudáveis (p < 0,001). Além disso, níveis séricos elevados de quemerina, uma adipocina inflamatória, correlacionaram-se positivamente com a CIMT (p < 0,05), indicando uma relação entre inflamação crônica, espessura arterial aumentada e maior carga de aterosclerose. Esses achados reforçam o papel do eco-Doppler na detecção precoce de placas de ateroma, que são precursores de eventos isquêmicos. A instabilidade dessas placas, associada à CIMT aumentada e a uma inflamação exacerbada, pode levar à sua ruptura, formação de trombos e isquemia cerebral. Assim, o eco-Doppler arterial é fundamental não apenas para diagnosticar aterosclerose, mas também para estratificar o risco de AVC isquêmico e direcionar intervenções preventivas mais eficazes^19^.

Além disso, o uso do eco-Doppler arterial pode ser complementado pelo uso de marcadores séricos para analisar a estabilidade da placa. A combinação do *duplex scan* com marcadores como metaloproteinase de matriz 9 (MMP-9), receptor tipo lectina para LDL oxidada (LOX-1) e glicoproteína de quina 40 (YKL-40) possibilita a identificação precoce de placas vulneráveis na artéria carótida em pacientes assintomáticos, apresentando uma sensibilidade de 87,67%, especificidade de 81,13% e acurácia diagnóstica de 84,92%. Assim, é possível identificar grupos com risco elevado para eventos cardiovasculares e cerebrovasculares^16,20^.

Os marcadores MMP-9, LOX-1 e YKL-40 são amplamente estudados na literatura como indicadores de instabilidade de placas ateroscleróticas. Seus níveis elevados têm sido consistentemente associados a um aumento no risco de ruptura das placas, sendo cada marcador representativo de processos biológicos específicos que contribuem para sua vulnerabilidade. A MMP-9 participa da degradação da matriz extracelular, enfraquecendo a capa fibrosa e permitindo a infiltração de células inflamatórias, o que aumenta a probabilidade de ruptura. O LOX-1, por sua vez, promove disfunção endotelial ao internalizar lipoproteínas oxidadas e desencadear respostas inflamatórias e apoptóticas, estando relacionado a placas hipoecoicas e isoecoicas detectáveis pelo *duplex scan*, características de vulnerabilidade estrutural. Já a YKL-40 (glicoproteína de quina 40) é uma proteína inflamatória envolvida na remodelação tecidual, cujos níveis elevados refletem um estado inflamatório crônico, vital para a desestabilização das placas e progressão aterosclerótica. Esses marcadores, ao indicar vulnerabilidade da estrutura das placas, permitem uma abordagem mais direcionada para estratificação de risco e intervenções terapêuticas^18^.

Geralmente, as carótidas com placas sintomáticas e grau significativo de oclusão apresentam uma escala de cinza menor e um índice de Kanbner elevado, que resulta da associação entre o grau de estenose, a mediana da escala de cinza e uma medida quantitativa das irregularidades da superfície, obtida por meio da imagem Doppler colorida e da ultrassonografia com contraste. Esses fatores são essenciais para a análise da vulnerabilidade da placa durante o eco-Doppler^21^. Isso possibilita, por meio da análise das imagens, a percepção de possíveis situações desfavoráveis, pois, à medida que a doença carotídea progride, tornam-se mais suscetíveis a desfechos como AVC isquêmico ipsilateral e os ataques isquêmicos transitórios^19^. Durante a análise realizada por ultrassonografia Doppler, observa-se que, conforme a estenose avança, maior é o fator de risco para o desenvolvimento de desfechos desfavoráveis correlacionados com eventos neurológicos (p = 0,001, OR = 8,9; IC95% 1,9-4,1)^22,23^.

Através da ultrassonografia Doppler, é possível analisar o movimento da placa carotídea. Em análises dinâmicas, o estudo das imagens demonstra que, durante a sístole e início da diástole, todos os componentes presentes na placa carotídea podem se mover na mesma direção, gerando um movimento concordante em algumas dessas placas. No entanto, em alguns casos, essa placa pode se mover em diferentes sentidos, gerando o chamado movimento discordante. Quando esse movimento é discordante, há indicação da presença de alto estresse da placa, que, quando quantificado, pode servir como uma medida indireta para a gravidade do risco de ruptura e, logicamente, para o desenvolvimento dos sintomas. A prevalência desse movimento discordante é de 89,1% em placas sintomáticas e de 17,9% em assintomáticas^9^.

Devido às características multifacetadas que as placas ateroscleróticas de carótida podem apresentar no ultrassom, ressalta-se a importância da avaliação multiparamétrica discutida até então para a tomada de decisão terapêutica adequada^21^. Avaliar os riscos de eventos indesejados, os custos envolvidos e a possibilidade de melhor qualidade de vida são elementos cruciais a serem considerados. Esses aspectos destacam a vantagem do Doppler em termos de custo-benefício para a avaliação das lesões ateroscleróticas, contribuindo, assim, para a identificação de pacientes com estenose sintomática e assintomática e auxiliando na decisão sobre a necessidade de endarterectomia carotídea, quando aplicável^24^.

A avaliação das placas ateroscleróticas na artéria carótida é essencial para prever riscos de doenças cardiovasculares. Embora o eco-Doppler seja amplamente utilizado por sua acessibilidade e capacidade de oferecer informações em tempo real, seu uso isolado pode limitar a detecção da diversidade de características das placas. Estudos destacam que métodos complementares, como ressonância magnética e tomografia computadorizada, são mais sensíveis para a identificação de componentes como hemorragia intraplaca, núcleo necrótico rico em lipídios e calcificações, todos importantes preditores de eventos cardiovasculares^25^. Além disso, técnicas avançadas como a tomografia por emissão de pósitrons permitem a visualização de processos inflamatórios, ampliando a compreensão da fisiopatologia das placas. A integração desses métodos oferece uma avaliação morfológica mais precisa, validada pela correlação com achados histológicos, fundamentando intervenções clínicas mais direcionadas e eficazes^15,24^.

Embora o presente estudo forneça uma análise abrangente da ultrassonografia Doppler na avaliação de placas ateroscleróticas, concentra-se exclusivamente na doença carotídea por meio dessa modalidade, sem considerar comparações aprofundadas com outras técnicas diagnósticas, como angiotomografia ou ressonância magnética. Ademais, a abordagem do presente estudo está limitada a revisões de literatura publicadas entre 2018 e 2024, o que restringe o escopo de análise.

Essa restrição temporal foi adotada com o objetivo de priorizar evidências recentes, refletindo os avanços mais atuais na prática clínica, nas tecnologias e nas abordagens terapêuticas relacionadas ao tema. Considerou-se que os últimos 7 anos representam um intervalo suficiente para captar tendências contemporâneas da literatura científica, alinhadas com as diretrizes mais recentes. No entanto, se reconhece que essa escolha pode ter excluído estudos clássicos ou com alta robustez metodológica publicados anteriormente, representando uma limitação da presente revisão.

Ainda que a presente revisão integrativa tenha conseguido um importante número de estudos com certa homogeneidade metodológica e temática, a realização de uma metanálise não foi conduzida devido à variabilidade nos desfechos avaliados, diferenças nos critérios diagnósticos, populações estudadas e métodos estatísticos empregados. Essa heterogeneidade comprometeria a validade estatística e clínica de uma análise quantitativa combinada. Optou-se, portanto, por uma síntese crítica, mais adequada ao perfil dos dados disponíveis.

O estudo traz uma contribuição pertinente para a prática do cirurgião vascular ao reforçar o papel do ultrassom Doppler como ferramenta diagnóstica na caracterização de placas ateroscleróticas carotídeas. Diferentemente das diretrizes da Sociedade Brasileira de Angiologia e Cirurgia Vascular e das diretrizes europeias, que enfatizam predominantemente o grau de estenose carotídea como principal critério para indicação de intervenção, este artigo amplia essa abordagem ao destacar a importância da análise multiparamétrica das placas ateroscleróticas, considerando características morfológicas como ecogenicidade e calcificações, além do uso de biomarcadores séricos (MMP-9, LOX-1 e YKL-40) para uma estratificação de risco mais precisa. Essa perspectiva sugere uma possível evolução nas recomendações futuras, incorporando critérios adicionais, além do percentual de estenose, para definir condutas terapêuticas de forma mais personalizada^26^.

## CONCLUSÃO

A ultrassonografia Doppler desempenha um papel essencial na avaliação das placas ateroscleróticas das artérias carótidas, permitindo a análise não invasiva de sua morfologia e a estimativa do risco de eventos cerebrovasculares, como o AVC. Sua análise detalhada destaca atributos específicos das placas, reforçando o valor do Doppler na estratificação de risco e na tomada de decisão clínica. Além disso, contribui significativamente ao destacar a relevância do *duplex scan* como abordagem custo-efetiva e prática, integrando o uso de marcadores séricos e análises multiparamétricas. Frente à literatura consagrada, o estudo reforça a importância do Doppler como ferramenta custo-efetiva e prática, auxiliando na tomada de decisão clínica e enriquecendo as estratégias de estratificação de risco na prática médica.

## Data Availability

Data included in the article/supplemental material: “All data generated or analyzed are included in this article and/or in the supplemental material”.
